# Spherical Fourier-Transform-Based Real-TimeNear-Field Shaping and Focusing in Beyond-5G Networks

**DOI:** 10.3390/s23063323

**Published:** 2023-03-22

**Authors:** Alessandro Felaco, Kamil Yavuz Kapusuz, Hendrik Rogier, Dries Vande Ginste

**Affiliations:** IDLab, Department of Information Technology, Ghent University-IMEC, Technologiepark-Zwijnaarde 126, 9052 Ghent, Belgium; kamilyavuz.kapusuz@ugent.be (K.Y.K.); hendrik.rogier@ugent.be (H.R.); dries.vandeginste@ugent.be (D.V.G.)

**Keywords:** array signal processing, beyond fifth-generation (B5G) wireless communication, holographic beamforming, multipole expansion, near-field focusing

## Abstract

For ultra-reliable high-data-rate communication, the beyond fifth generation (B5G) and the sixth generation (6G) wireless networks will heavily rely on beamforming, with mobile users often located in the radiative near-field of large antenna systems. Therefore, a novel approach to shape both the amplitude and phase of the electric near-field of any general antenna array topology is presented. Leveraging on the active element patterns generated by each antenna port, the beam synthesis capabilities of the array are exploited through Fourier analysis and spherical mode expansions. As a proof-of-concept, two different arrays are synthesized from the same active antenna element. These arrays are used to obtain 2D near-field patterns with sharp edges and a 30 dB difference between the fields’ magnitudes inside and outside the target regions. Various validation and application examples demonstrate the full control of the radiation in every direction, yielding optimal performance for the users in the focal zones, while significantly improving the management of the power density outside of them. Moreover, the advocated algorithm is very efficient, allowing for a fast, real-time modification and shaping of the array’s radiative near-field.

## 1. Introduction

Key performance indicators for the evolved fifth generation (E5G) and upcoming sixth generation (6G) mobile wireless communications networks promote continuous connection availability, strong reliability, huge device density and low air interface latency [1]. To unleash the full potential of E5G and 6G for indoor applications, a holistic multi-disciplinary approach is required based on disruptive communication technologies and innovative beamforming architectures [2,3,4]. In particular, to support extremely high data rates and to improve link reliability, advanced beamforming networks operating in millimeter-Wave (mmWave) and TeraHertz (THz) frequency bands play a key role. In such cases, however, the near-field (Fresnel) distance can amount to several dozens of meters. Therefore, the proper assumptions should be adopted to analyze the system’s performance, instead of the conventional far-field approach [5,6,7,8].

To enable antenna arrays to set up wireless communication in the Fresnel region, an appropriate focusing technique is essential to obtain properly shaped focal spots exhibiting high energy concentrations in the near-field region [9]. Moreover, to be able to modify the shape and position of focusing in real-time, the array’s antenna elements’ excitations require fast updates in terms of new amplitudes and phases. Therefore, the design of a practically feasible near-field focused array (NFFA) must fulfill stringent and sometimes conflicting requirements, often not occurring in standard base stations. On the one hand, the use of complex topologies or expensive materials must be prevented to reduce cost and footprint [10,11]. On the other hand, to achieve an improved wireless efficiency, minimization of power consumption and a reduction of the overall electromagnetic (EM) field emissions must be ensured [12,13].

This stimulated the development of a vast amount of numerical techniques, shaping these strong Fresnel-zone fields to concentrate elevated energy densities at one specific spot. These include methods based on the optimization of the power transmission efficiency (PTE) [14,15], energy-based models [16], gradient-based methods [17], target-field optimization techniques [18], far-field approximations [19,20,21], phase-conjugate [22] and quadratic-phase approximation methods [23]. While these methods are very time-efficient due to their simple nature, this also results in limited focusing capabilites. In multi-user communication, for instance, a better signal-to-interference ratio (SIR) must be provided by suppressing the radiation originating from undesired side lobes and secondary focal spots. In this context, multi-target focusing techniques adopt the one-focal-spot-per-user concept by exploiting the same time-frequency resources for increased throughput [24,25]. By making use of multi-target PTE [26], 2D time-reversal [27] or the angular spectrum projection method [28], these methods combine the results obtained by simultaneously solving several single-spot focusing problems over a number of target points. Decent results can be achieved in this way when carefully choosing the distance between the spots. These methods are, however, far from optimal due to the coherent interference between these basic shaped fields, which might result in detrimental secondary focal spots.

To ensure a proper simultaneous manipulation of the electric near-field’s levels over an entire 2D or 3D region of space, a full-fledged, field-shaping technique is required. This, however, often implies costly iterative optimizations or complex, impractical setups, which conflict with the rapid-response requirements of modern applications. Based on the alternate projection method [29], the work in [30] produces the required aperture field distribution by accurately placing and sizing the elements of a radial line slot array (RLSA). While its iterative component is optimized through an efficient in-house Method of Moment (MoM), it still requires a modification of the array structure to obtain different target shapes. Alternatively, the optimized multi-target time-reversal (O-mt-TR) technique proposed in [31,32], accurately models the characteristics of the simulation domain by means of probing. This method pays a high price in terms of computational complexity, with simulations taking several hours. In order to bypass the sensing step, the work in [33] suggests a technique based on the linear sampling method (LSM) to shape the field intensity within an unknown scenario. In this case, however, due to the ill-posedness of the problem, the procedure becomes less computationally effective as the number of control points grows. Hence, the development of a new, efficient tool to rapidly synthesize the excitations required to shape the radiated power in the near-field of an antenna aperture constitutes a timely and highly relevant research challenge.

In this paper, a novel method is presented, based on active far-field radiation pattern data, to determine the complex current excitations required to shape the electric near-field produced by general antenna arrays. The proposed method constitutes an important extension of the conventional far-field beam-shaping technique, enabling the generation of arbitrary field shapes on a sphere located in the array’s radiative near-field. Specifically, the amplitude and phase of the electric field radiated by the array are controlled on a spherical surface centered at the array’s phase center and with a radius such that the surface is located in the array’s radiative near-field. The novel technique smoothly transitions between near- and far-field shaping by modifying the radius of said sphere. In contrast to [34], the input 2D field data is highly compressed through a spherical Fourier transform and then used to build an equivalent system matrix. After this one-time set-up operation, and owing to compressed multidimensional data in the Fourier domain, the advocated method provides a direct optimal solution in real-time to any near-field shaping problem in a fraction of a second, bypassing lengthy iteration procedures or side lobes optimizations.

This paper is organized as follows. Section 2 outlines the problem statement, details the far-to-near-field transformation and the subsequent shaping of the fields radiated by the array aperture’s current distribution, based solely on the compressed active far-field data. Section 3 validates and demonstrates the technique through both numerical and full-wave experiments. Conclusions and future plans are summarized in Section 4.

## 2. Theory

### 2.1. Problem Statement

**Figure 1:**
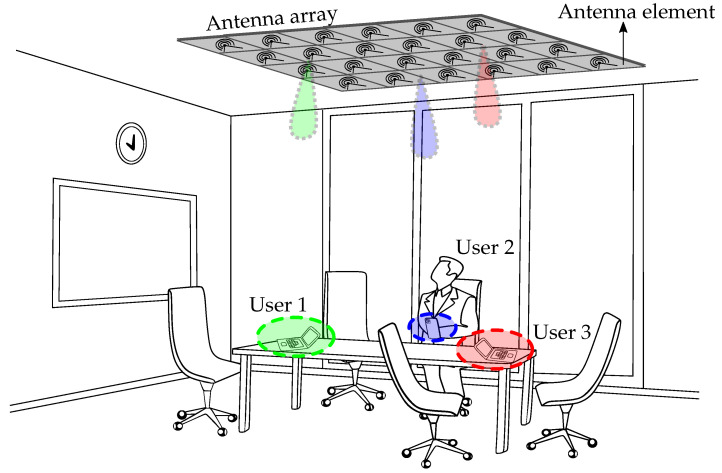
Application scenario. Users are distributed in the radiative near-field of the antenna array. Each user is targeted by an individual focal spot to ensure the best quality of communication.

Consider the application scenario of Figure 1, where a number of users are distributed in the radiative near-field of an antenna array. The goal of this work is to determine the complex excitation currents required to shape the array’s electric near-field E(r) in order to ensure user-specific and quasi interference-free operation.

**Figure 2:**
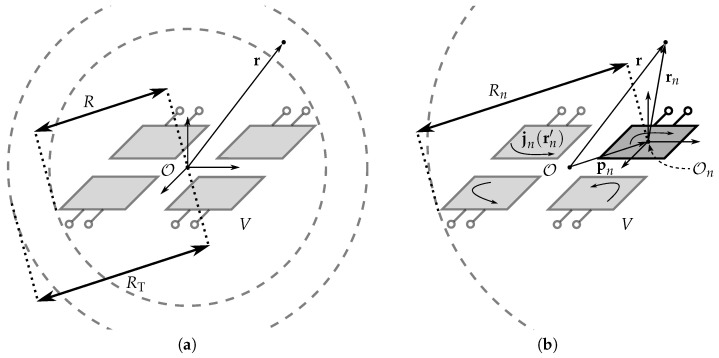
(**a**) Conceptual sketch of an array of volume *V* circumscribed by a sphere of radius *R* and with its phase center coinciding with the origin of the coordinate system O(x,y,z). The goal is to shape the array’s emitted field so that it corresponds to a desired pattern on a sphere with radius $R_{T}$. (**b**) Each nth array element, fed through the nth port, is attached to a local phase center, indicated by a vector $p_{n}$, and coinciding with a local coordinate system’s origin $O_{n}\left(x_{n},y_{n},z_{n}\right)$. The circumscribing sphere with regard to the local coordinate system has a radius $R_{n}$. A current $I_{n}$ is injected into the nth port (see also Figure 3), generating a current distribution $j_{n}$ on the entire array.

The array is conceptually represented in Figure 2a. Attached to its phase center is the origin of the *global* coordinate system O(x,y,z), which also coincides with the center of the array’s circumscribing sphere of radius *R*. In the following sections, E(r) is shaped so that it corresponds to a prescribed target-field distribution $T(R_{T}\hat{r})$ over the surface of a sphere of radius $R_{T}$ ≥ *R* (Figure 2a), where $\hat{r}$ denotes the radial unit vector. Therefore, we propose a novel algorithm that efficiently computes the required excitations of the array’s antenna elements, using only their active radiation patterns as input.

### 2.2. Far-to-Near-Field Transformation

**Figure 3:**
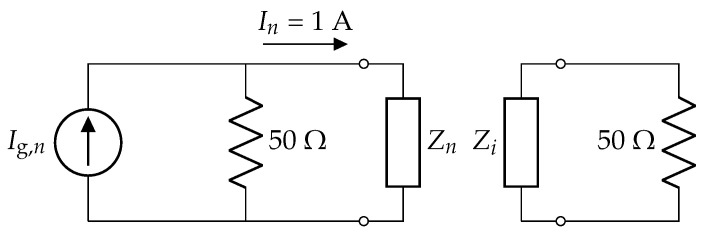
Excitation of the nth active far-field radiation pattern. The nth port is excited by a Norton equivalent source formed by a current source $I_{g,n}$ in parallel with a 50 Ω resistance. The source is chosen such that a current $I_{n}$ = 1 A is injected into the nth antenna, represented by its input impedance $Z_{n}$. All other ports for *i*≠*n* are instead terminated by a 50 Ω resistor.

Consider the nth antenna element with its phase center indicated by the vector $p_{n}$ and attached to the origin of a *local* coordinate system $O_{n}\left(x_{n},y_{n},z_{n}\right)$ (Figure 2b). To obtain the nth active far-field radiation pattern, this element is excited via its port by a unit current $I_{n}$ = 1 A injected by an appropriate Norton equivalent source $I_{g,n}$. All other antenna elements *i* ≠ *n* are terminated by a 50 Ω impedance. This is schematically shown in Figure 3. Due to mutual coupling, the excitation gives rise to a current distribution $j_{n}\left(r_{n}^{\prime }\right)$, flowing over the whole volume *V*, i.e., on all antenna elements in the array, where $r_{n}^{\prime }$ is defined in the local coordinate system $O_{n}$.

The electric field $E_{n}\left(r_{n}\right)$, generated by the current density $j_{n}\left(r_{n}^{\prime }\right)$ and defined with respect to $O_{n}$ is then obtained by the electric field integral equation (EFIE) [35], which reads

(1) $$E_{n}\left(r_{n}\right)=-j\omega \mu \int_{V}G\left(r_{n}^{},r_{n}^{\prime }\right)\cdot j_{n}\left(r_{n}^{\prime }\right)dr_{n}^{\prime },$$

where ω is the angular frequency, and μ is the free-space permeability. In (1), we make use of a multipole expansion for the dyadic Green’s function G [36] detailed in Appendix A and valid for $r_{n}$ = $r_{n}$ ≥ $R_{n}$, i.e.,

(2) $$G\left(r_{n}^{},r_{n}^{\prime }\right)=-\frac{jk}{4\pi }\sum_{l,m}^{\infty }H_{l}^{m}\left(kr_{n}\right){∯}_{\Omega }\left[I-\hat{k}\hat{k}\right]e^{jk\cdot r_{n}^{\prime }}{\bar{Y}}_{l}^{m}\left(\hat{k}\right)d\hat{k},$$

where $R_{n}$ (Figure 2b) is the radius of the sphere circumscribing the current distribution $j_{n}\left(r_{n}^{\prime }\right)$ and thus the antenna array, measured with respect to $O_{n}$. Further, *k* is the wavenumber, I represents the unit dyadic, and ${\bar{Y}}_{l}^{m}$ is the complex conjugate of the $l^{\mathrm{th}}$ order, $m^{\mathrm{th}}$ degree scalar spherical harmonic [37]. The multipole function $H_{l}^{m}$ in (2) is defined as

(3) $$H_{l}^{m}\left(kr_{n}\right)=j^{-l}h_{l}^{\left(2\right)}\left(kr_{n}\right)Y_{l}^{m}\left({\hat{r}}_{n}\right),$$

where $h_{l}^{\left(2\right)}$ is the $l^{\mathrm{th}}$ order spherical Hankel function of the second kind ([38], p. 437). Furthermore, throughout this Section, we make use of the following notation,

(4) $$\sum_{l,m}^{\infty }=\sum_{l=0}^{\infty }\sum_{m=-l}^{l}.$$

Substituting (2) into (1) yields a compact multipole expansion of the electric field $E_{n}$, valid for $r_{n}$ ≥ $R_{n}$ and thus including the array’s radiative near-field, as

(5) $$E_{n}\left(r_{n}\right)=-jk\sum_{l,m}^{\infty }f_{l,n}^{m}H_{l}^{m}\left(kr_{n}\right),$$

where the coefficients $f_{l,n}^{m}$ are the result of a spherical Fourier transform of the nth current-normalized and active far-field radiation pattern $F_{n}$, as ([39], Equation (8))

(6) $$f_{l,n}^{m}={∯}_{\Omega }F_{n}\left(\hat{k}\right){\bar{Y}}_{l}^{m}\left(\hat{k}\right)d\hat{k},$$

(7) $$\begin{array}{cc} F_{n}\left(\hat{k}\right) & =-\frac{j\omega \mu }{4\pi }\left[I-\hat{k}\hat{k}\right]\cdot \int_{V}e^{jk\cdot r_{n}^{\prime }}j_{n}\left(r_{n}^{\prime }\right)dr_{n}^{\prime }. \end{array}$$

As explained in [39], when a far-field is expressed in the spherical basis as F = $F_{\theta }\hat{\theta }$ + $F_{\phi }\hat{\phi }$, both $F_{\theta }$ and $F_{\phi }$ are not continuous functions on the unit sphere Ω. In particular, they exhibit discontinuities at the north and south poles. Therefore, to compute (6), we express the radiation pattern in Cartesian components as F = $F_{x}\hat{x}$ + $F_{y}\hat{y}$ + $F_{z}\hat{z}$ [40]. These radiation patterns are, for instance, obtained via simulations or measurements.

Further, it is known that each $F_{n}$ is (quasi-)band-limited, meaning that it can be efficiently described using an $L_{n}$-order spherical Fourier transform, as ([39], Equation (9))

(8) $$\begin{array}{cc} F_{n}\left(\hat{k}\right) & \approx \sum_{l,m}^{L_{n}}f_{l,n}^{m}Y_{l}^{m}\left(\hat{k}\right), \end{array}$$

(9) $$\begin{array}{cc} \epsilon (L_{n}) & =20\log_{10}\left(\max_{\hat{k}}\left|F_{n}\left(\hat{k}\right)-\sum_{l,m}^{L_{n}}f_{l,n}^{m}Y_{l}^{m}\left(\hat{k}\right)\right|\right), \end{array}$$

where $\epsilon (L_{n})$ is the maximum absolute error in dBV stemming from (8). To minimize $\epsilon (L_{n})$, on the one hand, the $f_{l,n}^{m}$ coefficients (6) are computed through a *Q*-order Lebedev quadrature [41]. The value of *Q* yielding the best performance is obtained by rounding up $L_{n}$ to the closest available quadrature order. On the other hand, for a radiating structure with a radius $R_{n}$, modes of order $l>kR_{n}$ provide little additional information about its radiation pattern and electric field [42]. As a rule of thumb, both the summations in (5) and (8) may therefore be truncated at

(10) $$L_{n}=⎣kR_{n}⎦.$$

Whereas a higher value of $L_{n}$ might reduce ϵ, a trade-off between accuracy and performance must be carefully considered. Empirically, it was verified that a value of maximally ϵ < 10 dBV is required to accurately model the device under test. To speed up this computation, the values that the function ${\bar{Y}}_{l}^{m}\left(\hat{r}\right)$ in (6) assumes for all possible Lebedev quadrature nodes are stored during the set-up phase of the algorithm, amounting to circa 100 MB of data, and used for future simulations.

### 2.3. Phase Center Translation

Owing to linearity, the total electric field E(r) produced by the array is given by the weighted sum of the electric fields produced by all *N* antenna elements, as in

(11) $$E\left(r\right)=\sum_{n=1}^{N}I_{n}E_{n}\left(r\right),$$

where $I_{n}$ is the nth feeding current, and $E_{n}\left(r\right)$ is the electric field produced by exciting the nth antenna element, expressed with respect to the global phase center O. To perform a translation from the nth local to the global coordinate system, we make use of some simple vector algebra:

(12) $$E_{n}\left(r_{n}\right)=E_{n}\left(r-p_{n}\right)=-jk\sum_{l,m}^{L_{n}}f_{l,n}^{m}H_{l}^{m}\left(k\left(r-p_{n}\right)\right),$$

where $p_{n}$ (Figure 2b) is the vector indicating the position of the nth local phase center in the global coordinate system. An addition theorem for (12) is easily derived from the classic theorem for spherical Hankel functions ([43], Equation (6)). For r = *r* ≥ *R* ≥ $p_{n}$, we have

(13) $$H_{l}^{m}\left(k\left(r-p_{n}\right)\right)=\sum_{\lambda ,\mu }^{\Lambda_{n}}T_{\lambda l}^{\mu m}\left(kp_{n}\right)H_{\lambda }^{\mu }\left(kr\right),$$

where the translation operator $T_{\lambda l}^{\mu m}$ is defined as

(14) $$T_{\lambda l}^{\mu m}\left(kp_{n}\right)=4\pi {\left(-1\right)}^{\lambda -l+\mu }\sum_{q=|l-\lambda |}^{l+\lambda }G\left(l,m,\lambda ,-\mu ,q\right)\;J_{q}^{m-\mu }\left(kp_{n}\right),$$

in which the symbol G indicates a Gaunt coefficient ([44], Equation (A-2)) and where

(15) $$J_{q}^{m-\mu }\left(kp_{n}\right)=j^{-q}j_{q}\left(kp_{n}\right)Y_{q}^{m-\mu }\left({\hat{p}}_{n}\right),$$

with $j_{q}$ being the qth order spherical Bessel function of the first kind. Plugging (13) into (12), we express the nth global electric field, valid for r≥R, as

(16) $$\begin{array}{cc} E_{n}\left(r\right) & =-jk\sum_{\lambda ,\mu }^{\Lambda_{n}}f_{\lambda ,n}^{\mu }H_{\lambda }^{\mu }\left(kr\right), \end{array}$$

(17) $$\begin{array}{cc} f_{\lambda ,n}^{\mu } & =\sum_{l,m}^{L_{n}}T_{\lambda l}^{\mu m}\left(kp_{n}\right)f_{l,n}^{m}, \end{array}$$

where (17) is a linear mapping that transforms the original set of coefficients $f_{l,n}^{m}$, determined up to order $L_{n}$ and with respect to the nth local coordinate system $O_{n}\left(x_{n},y_{n},z_{n}\right)$, into the translated coefficients $f_{\lambda ,n}^{\mu }$ required up to order $\Lambda_{n}$ and with respect to the global coordinate system O(x,y,z).

To determine the optimal value of $\Lambda_{n}$, we make use of the nth *cumulative power spectrum* $\Gamma_{n}$, defined as ([39], Equation (23))

(18) $$\Gamma_{n}\left(L_{n}\right)=\sum_{l,m}^{L_{n}}{\left|f_{l,n}^{m}\right|}^{2},$$

which is a measure for the power radiated when exciting the nth antenna element. We exploit the property that, under translation of the antenna’s phase center, the cumulative power spectrum remains constant. Theoretically, $\Lambda_{n}$ can be obtained from the following equivalence

(19) $$\Gamma_{n}\left(L_{n}\right)=\sum_{l,m}^{L_{n}}|f_{l,n}^{m}{|}^{2}=\sum_{\lambda ,\mu }^{\Lambda_{n}}{\left|f_{\lambda ,n}^{\mu }\right|}^{2}=\Gamma_{n}\left(\Lambda_{n}\right).$$

**Figure 4:**
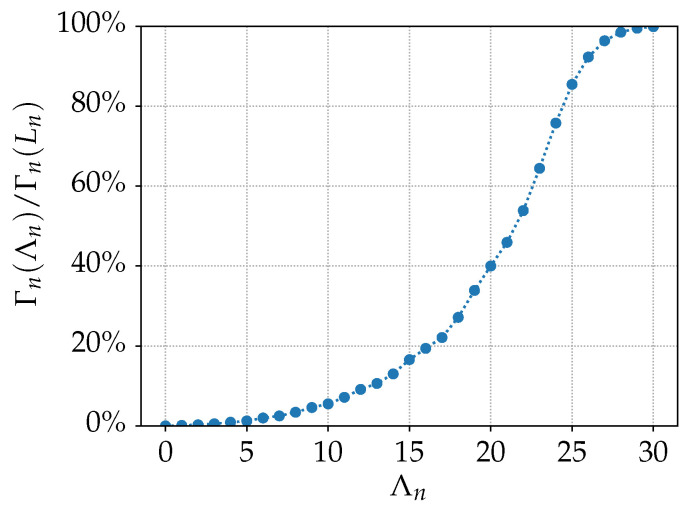
Typical sigmoid behavior of the cumulative power spectra ratio $\Gamma_{n}\left(\Lambda_{n}\right)/\Gamma_{n}\left(L_{n}\right)$ for increasing $\Lambda_{n}$. A sufficient value for $\Lambda_{n}$ is reached when this ratio is at least 99%.

Numerically, we compute $\Lambda_{n}$ iteratively, since the new cumulative power spectrum $\Gamma_{n}\left(\Lambda_{n}\right)$ approaches $\Gamma_{n}\left(L_{n}\right)$ asymptotically for increasing $\Lambda_{n}$, as shown in Figure 4. Simulations have shown that defining $\Lambda_{n}$ as the value for which $\Gamma_{n}\left(\Lambda_{n}\right)$ is at least 99% of $\Gamma_{n}\left(L_{n}\right)$ yields the best balance between computational time and accuracy.

### 2.4. Near-Field Intensity Shaping

Assume that the antenna array is designed to emit an electric field with a specific polarization $\hat{u}$. We split each nth electric field $E_{n}$ as

(20) $$\begin{array}{cc} E_{n}\left(r\right) & =E_{n}^{\mathrm{co}}\left(r\right)\hat{u}+E_{n}^{\mathrm{cross}}\left(r\right), \end{array}$$

(21) $$\begin{array}{cc} E_{n}^{\mathrm{co}}\left(r\right) & =E_{n}\left(r\right)\cdot \hat{u}, \end{array}$$

(22) $$\begin{array}{cc} E_{n}^{\mathrm{cross}}\left(r\right) & =E_{n}\left(r\right)-E_{n}^{\mathrm{co}}\left(r\right)\hat{u}, \end{array}$$

so that by combining (11), (16) and (21), the desired co-polar component of the array’s electric field values, at a distance $R_{T}$ from the array’s phase center, i.e., for r = $R_{T}\hat{r}$, is given by

(23) $$E^{\mathrm{co}}\left(R_{T}\hat{r}\right)=-jk\sum_{n=1}^{N}I_{n}\sum_{\lambda ,\mu }^{\Lambda }f_{\lambda ,n}^{\mu ,\mathrm{co}}H_{\lambda }^{\mu }\left(kR_{T}\hat{r}\right),$$

where $f_{\lambda ,n}^{\mu ,\mathrm{co}}$ = $f_{\lambda ,n}^{\mu }\cdot \hat{u}$, and Λ = $\max\left(\Lambda_{n}\right)$. To achieve near-field shaping, we set the left-hand side of (23) equal to a target field $T(R_{T}\hat{r})$ = $T\left(R_{T}\hat{r}\right)\cdot \hat{u}$. Subsequently, the currents $I_{n}$ that best approximate the desired pattern are obtained as

(24) $$-jk\sum_{n=1}^{N}I_{n}\sum_{\lambda ,\mu }^{\Lambda }f_{\lambda ,n}^{\mu ,\mathrm{co}}H_{\lambda }^{\mu }\left(kR_{T}\hat{r}\right)\approx T\left(R_{T}\hat{r}\right).$$

It is important to note that the only restriction imposed by the addition theorem is that $R_{T}$ ≥ *R*, meaning that it is possible to obtain the values $T(R_{T}\hat{r})$ on the surface of a sphere located entirely in the array’s radiative near-field. Combining (3) and (24) and performing a Λ-order multipoles expansion (8) of $T(R_{T}\hat{r})$ yields

(25) $$\sum_{n=1}^{N}I_{n}f_{\lambda ,n}^{\mu ,\mathrm{co}}=\frac{{∯}_{\Omega }T\left(R_{T}\hat{r}\right){\bar{Y}}_{\lambda }^{\mu }\left(\hat{r}\right)d\hat{r}}{j^{-\lambda +1}kh_{\lambda }^{\left(2\right)}\left(kR_{T}\right)}\overset{\Delta }{=}t_{\lambda }^{\mu }.$$

For very large values of $R_{T}$, the advocated method smoothly transitions into a more refined version of the traditional far-field beamforming technique. This is easily proven by calculating the denominator in (25) for large arguments. Employing the identity ([38], Equation (10.1.17)) yields

(26) limRT→∞j−λ+1khλ(2)(kRT)=limRT→∞−e−jkRTRT∑n=0λλ+0.5n2jkRT−n≈−e−jkRTRT,

which is equivalent to a simple change of overall amplitude and phase reference of $T(R_{T}\hat{r})$. In this way, for very large $R_{T}$ values, the advocated method translates to a far-field shaping technique.

Finally, we collect the sought-after excitation currents $I_{n}$ into the unknown vector i and the target Fourier coefficients $t_{\lambda }^{\mu }$ into the known vector t. This way, we cast (25) into the following linear system of equations,

(27) M·i=t,

where the elements of the system matrix M are the antenna element’s coefficients $f_{\lambda ,n}^{\mu ,\mathrm{co}}$. As a result, M has dimensions M×N, where $M={\left(\Lambda +1\right)}^{2}$ is the number of harmonics involved in the computation, and *N* is the amount of antennas in the array. Before solving (27), however, we have to verify that the problem is well-conditioned. This is equivalent to the columns (or rows) of M being linearly independent and, as a consequence, a limited condition number κ(M).

The nth column of M collects the translated spherical Fourier spectrum’s coefficients of the nth antenna element’s active radiation pattern (17). Therefore, the elements of M are only a function of the original active radiation patterns and of their respective element positions $p_{n}$. In general, for antenna arrays consisting of identical elements with sufficient inter-element spacing (i.e., several λ), the difference between all the active radiation patterns is practically negligible. In this scenario, κ(M) becomes solely a function of the array layout. In other words, by ensuring that the antennas are spaced sufficiently far from each other, the columns of M will be sufficiently linearly independent. For denser antenna arrays, it was empirically verified that a spacing of at least λ/3 yields values of κ(M) in the order of $10^{3}∼10^{4}$, while a spacing of λ/2 reduces it further to order $10^{2}∼10^{3}$. For very dense antenna arrays with a spacing smaller than λ/3, the problem of an increasing condition number is partially mitigated by the higher coupling between the antenna elements. Owing to the asymmetries in the array layout, a larger variation in the data is introduced, reducing the correlation between the rows of M. While in this case the set-up time would inevitably increase, the novel method is still applicable to very dense arrays owing to this set-up operation (i.e., obtaining the active far-field data and computing M) being performed only once per layout. However, special care should be taken when interpreting this superdirective solution, as it might be very sensitive to small changes in array geometry, excitations and operating frequency. On the other hand, it is known from the literature [45] that the value of Λ increases linearly with the array size. Since the value of *M*, and therefore the number of rows of M, scale quadratically with Λ, it is generally the case that M≫N, meaning that (27) corresponds to an overdetermined linear system. Since we can ensure that M has full column rank, we opt for a least-squares solution as

(28) $$\begin{array}{cc} i & =M^{+}\cdot t, \end{array}$$

(29) $$\begin{array}{cc} M^{+} & ={\left(M^{†}M\right)}^{-1}M^{†}, \end{array}$$

where † indicates the conjugate transpose operation. Note that the construction of the system matrix M and the subsequent computation of its pseudo-inverse $M^{+}$ are part of the set-up process of the algorithm. Once $M^{+}$ has been determined for a specific array, the process of adjusting the target in both distance and shape and obtaining the necessary feeding currents simply boils down to two simple steps that can be performed in real time. First, the target vector t is determined via (25) by a Λ-order Lebedev numerical quadrature, requiring circa Λ samples. Its evaluation requires 2Λ multiplications and Λ additions. For all orders λ and degrees μ, the construction of t needs $3\Lambda {\left(\Lambda +1\right)}^{2}\approx 3\Lambda^{3}$ operations. Second, one matrix–vector multiplication is performed to evaluate (28). Since the pseudo-inverse matrix $M^{+}$ has dimensions N×M, while the vector t has dimensions M×1, a total of 2NM operations are required. Since $M={\left(\Lambda +1\right)}^{2}$, the total number of operations scales as $2N\Lambda^{2}$. Therefore, the near-field shaping procedure is performed in less than a second, rendering the proposed method suitable for real-time field-shaping applications.

## 3. Validation

### 3.1. Numerical Validation

To validate the novel method, we make use of an *x*-polarized half-wave dipole antenna of length 136 mm, 0.9 mm wire diameter and operating at the frequency of 1 GHz (λ = 30 cm) as the elementary antenna element in our array configurations. A planar array consisting of *N* = 197 such elements is then synthesized. The antennas are distributed inside a circular region of radius *R* = 4λ = 1.2 m, forming a grid with inter-element spacing of λ/2 = 15 cm. In this experiment, we choose the radius of the target sphere as $R_{T}$ = 6λ = 1.8 m. As this size amounts to approximately 75% of the array’s aperture dimension, the shaping process takes place on a sphere in the radiative near-field of the structure.

**Figure 5:**
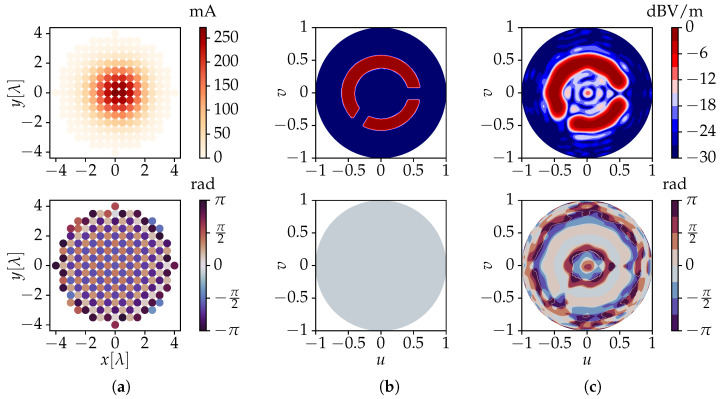
Results for a synthesized planar array consisting of 197 *x*-polarized dipoles. The figures show the amplitudes (**top**) and phases (**bottom**) of (**a**) the feeding currents obtained by the novel algorithm, for a (**b**) circle-like shaped target near-field and (**c**) the co-polar electric field solution obtained by the novel method. The results in (**b**,**c**) are drawn for the upper hemisphere of a target sphere of radius $R_{T}$ = 6λ, where u=sinθcosϕ and v=sinθsinϕ.

**Figure 6:**
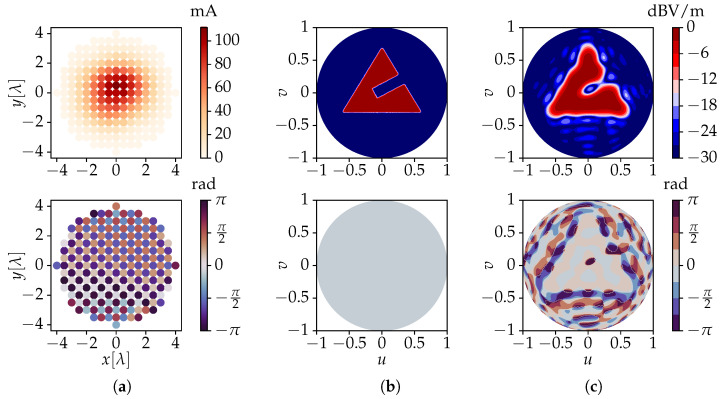
Results for a synthesized planar array consisting of 197 *x*-polarized dipoles. The figures show the amplitudes (**top**) and phases (**bottom**) of (**a**) the feeding currents obtained by the novel algorithm, for a (**b**) triangle-like shaped target near-field and (**c**) the co-polar electric field solution obtained by the novel method. The results in (**b**,**c**) are drawn for the upper hemisphere of a target sphere of radius $R_{T}$ = 6λ, where u=sinθcosϕ and v=sinθsinϕ.

The theory described in Section 2 dictates that each of the 197 active radiation patterns $F_{n}$ is to be determined, which represents quite a cumbersome task. However, owing to the rather large inter-element distance, the mutual coupling between neighboring antenna elements remains quite limited. Therefore, we may approximate all $F_{n}$ by the active radiation pattern $F_{0}$ of the element closest to the array’s center, i.e., the one located at *x* = *y* = 0. This is obtained via a full-wave simulation performed by the frequency domain solver of *CST Microwave Studio* [46] and normalized according to Figure 3. These data are used as input for a *Python* [47] script implementing the advocated algorithm. The spherical Fourier transform of $F_{0}$ is computed via (6) with an accuracy of −10 dB, requiring a value of $L_{0}$ = 18. Using (17), the resulting $f_{l,0}^{m}$ coefficients are then translated to the 197 different antenna positions. According to (19), a value of Λ = $\max\left(\Lambda_{n}\right)$ = 30 is needed, corresponding to *M* = ${\left(\Lambda +1\right)}^{2}$ = 961 harmonics involved in the numerical computation. This results in a 961 × 197 system matrix M, with a condition number κ(M) ≈ 3547. Subsequently, the target’s spherical Fourier transform and its corresponding target coefficients t are determined via (25). Finally, the unknown vector i is obtained via (28). This process required only 230 ms on an Intel Core i7-8650U processor running at 1.90 GHz with 16 GB of memory. Two examples are shown in Figure 5 and Figure 6, where the plots’ labels are given by

(30) $$\begin{array}{cc} u & =\sin\theta \cos\phi , \end{array}$$

(31) $$\begin{array}{cc} v & =\sin\theta \sin\phi , \end{array}$$

where θ ∈ $\left[0,\frac{\pi }{2}\right]$ and ϕ ∈ $\left[0,2\pi \right]$. In Figure 5a and Figure 6a, the required excitation currents for all 197 antennas computed by the novel method for the two target fields are shown.

The current amplitudes produced by the advocated algorithm naturally exhibit a Gaussian-like distribution, with elements closer to the array’s center receiving higher excitations. This is typical of tapering techniques [48], which are usually required in traditional array focusing methods as an additional step to reduce side-lobe levels. On the other hand, the current phases exhibit a checkerboard-like pattern, with neighboring elements having close to perfectly opposing phases. This is required to achieve radiationless interference between adjacent elements, allowing the remaining evanescent fields to carry shape features to the target surface [34]. The corresponding co-polar electric near-fields’ values obtained for the upper hemisphere of the target surface are shown in Figure 5c and Figure 6c.

A very close resemblance to the target fields of Figure 5b and Figure 6b, which are shapes with sharp edges, is obtained. While at first sight the phase profile is not overall flat, the novel method ensures the correct values over the areas of interest, i.e., where the shapes are defined. Note that the phase values are irrelevant in areas where the magnitudes of the field values are very low. Since the advocated algorithm operates in the Fourier domain, where a large amount of far-field data is replaced by a compact angular spectrum description, it requires only about 100 ms to import any new target shape and then determine the currents necessary to obtain it. As previously mentioned, our novel method is therefore applicable to real-time field-shaping.

### 3.2. Full-Wave Validation and Application

**Figure 7:**
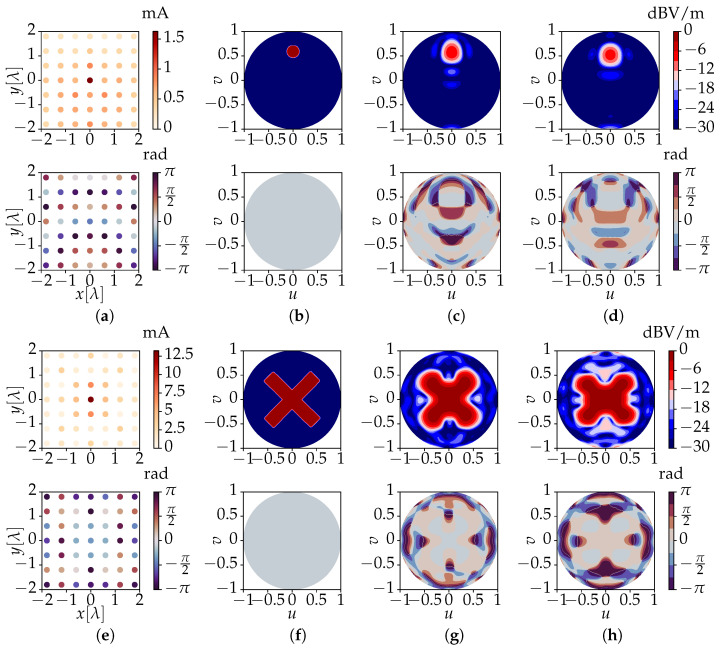
Results for a 7 × 7 planar array consisting of 49 *x*-polarized dipoles. The figures show the amplitudes (**top**) and phases (**bottom**) of (**a**,**e**) two sets of feeding currents obtained by the novel algorithm corresponding to (**b**,**f**) two differently shaped target near-fields. Further, we have (**c**,**g**) the co-polar electric field solutions obtained by the novel method and (**d**,**h**) the co-polar near-fields resulting from a full-wave simulation of the 7 × 7 array, excited by the currents in (**a**) and (**e**), respectively. The results are drawn for the upper hemisphere of a target sphere of radius $R_{T}$ = 4λ, where u=sinθcosϕ and v=sinθsinϕ.

To further validate the method, the results of the synthesis using the advocated technique are compared to accurate full-wave simulations using CST Microwave Studio. To limit the simulation time in CST, we design a 7 × 7 planar array using the same dipole antenna as antenna element. The inter-element spacing is 0.6λ = 18 cm, for a total size of circa 121 × 108 cm${}^{2}$. The radius of the target sphere is now $R_{T}$ = 4λ = 1.2 m, corresponding to one array aperture, to guarantee that the shaping process happens on a sphere located in the radiative near-field. The target fields are shown in Figure 7b,f. As in the previous section, the $f_{l,0}^{m}$ coefficients pertaining to the center antenna element are processed by (17), yielding a value of Λ = $\max\left(\Lambda_{n}\right)$ = 22. This corresponds to *M* = ${\left(\Lambda +1\right)}^{2}$ = 529 harmonics involved in the numerical computation, resulting in a 529 × 49 system matrix M, with a condition number κ(M) ≈ 3. Computing the required currents for this array took circa 160 ms.

**Figure 8:**
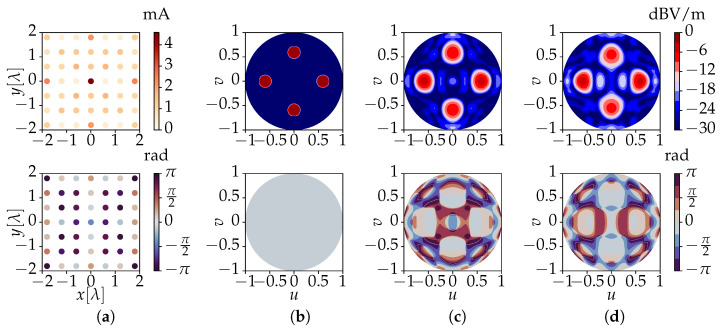
Results for a 7 × 7 planar array consisting of 49 *x*-polarized dipoles. The figures show the amplitudes (**top**) and phases (**bottom**) of (**a**) the feeding currents obtained by the novel algorithm corresponding to (**b**) four separate focal spots. Further, we have (**c**) the co-polar electric field solutions obtained by the novel method and (**d**) the co-polar near-fields resulting from a full-wave simulation of the 7 × 7 array, excited by the currents in (**a**). The results are drawn for the upper hemisphere of a target sphere of radius $R_{T}$ = 4λ, where u=sinθcosϕ and v=sinθsinϕ.

Figure 7a,e show the feed currents that are computed by means of the novel method in order to obtain the target fields. The resulting field values are shown in Figure 7c,g. We now also use the computed currents to feed the array in CST Microwave Studio and perform a full-wave simulation of the array’s resulting fields. These are shown in Figure 7d,h. Finally, we return to the application scenario of Figure 1, where *U* users are located close to one another. To ensure a good coverage for each individual user, the currents (and resulting electric near-field) obtained from the experiment of Figure 7a are combined in order to focus the power separately at *U* different spots. Furthermore, through a proper implementation of the feeding architecture of the array system, this permits us to deliver *U* different data streams to the users. The outcome of such a scenario, with *U* = 4, is shown in Figure 8.

When comparing target patterns, numerical solutions and full-wave results, an excellent agreement is observed, in both amplitude and phase. Any small difference is due to the approximation made by employing the center element active radiation pattern to represent all the remaining elements. This choice is, however, justified by both the enormous amount of time saved in performing the simulations of the antenna patterns and by the very good match between the numerical results and the full-wave simulations. Note that compared to the previous section, there is a loss of resolution as the sharp edges of the target fields are less accurately reproduced. Nevertheless, this is merely due to the use of a smaller array aperture rather than to the algorithm’s loss of accuracy.

### 3.3. Discussion on Computational Efficiency

All the methods referenced in this work, including the presented technique, require a one-time set-up operation. While an accurate description of this set up is not always provided by the respective authors, it can be estimated to be approximately the same across all algorithms. Therefore, we focus the following comparison on the time required for the actual electric near-field shaping process across different methods:

- Novel method: The number of operations required is approximately $3\Lambda^{3}$, where Λ is the chosen order of the spherical Fourier transform. For the largest array studied, with *N* = 200 and Λ = 30, the shaping procedure required around 200 ms and circa 100 MB of pre-computed data.
- O-mt-TR [31]: The amount of operations required is of order $M^{L}$, where *M* are the variables to optimize, and *L* is the amount of control points used. As the computational time scales exponentially with *L*, several hours are required to achieve shaping, hence excluding this algorithm from real-time applications. A variation of this technique, being O-mt-LSM [33], does not require any set-up phase, but this results in reduced computational efficiency and resolution.
- Smart skin holography [13]: While a quite good resolution is obtained in near-field shaping, the process required circa 20 min per shape. Again, this algorithm is also not suited to real-time applications.
- Method of Moments (MoM) [30]: A radial slot array is synthesised with an in-house MoM code and tailored for a specific target shape. The amount of time usually required to fill an MoM matrix and solve the matrix system is not compatible with real-time applications.
- Angular spectrum projection method [28]: This method’s efficiency is quantified as largely faster than the O-mt-TR. At the same time, the obtained target shapes exhibit a very poor resolution and are obtained as a discontinuous collection of discrete points.

Therefore, we conclude that when compared to other available near-field shaping methods, the technique proposed in this work excels in terms of computational efficiency, resolution and suitability. Furthermore, in contrast to most state-of-the-art literature, an accurate quantification of the number of operations, computational time and memory usage were provided.

## 4. Conclusions

A novel algorithm was developed to obtain near-field intensity shaping through general antenna array topologies, outperforming traditional techniques. The method leverages the simulated active far-field radiation patterns of the antenna elements. By making use of a spherical Fourier transform and multipole expansion of these radiation patterns, the advocated algorithm performs a far-to-near-field transform to quickly determine the amplitude and phases required to shape the co-polar component of the electromagnetic field produced by general array topologies. Specifically, and as an extension of conventional far-field beam-shaping techniques, the proposed algorithm achieves an accurate shaping over the surface of a sphere with a prescribed radius located in the array’s radiative near-field region. The novel method produces sharp patterns, while consuming a very small amount of resources, making it suitable for real-time operation.

As a proof-of-concept, 197-dipoles arrays were exploited to implement near-field focusing for two different intricate shapes. The undesired secondary beams within the target region were significantly suppressed by around 30 dB. Furthermore, intensity-shaping with smaller arrays, comprising 49 dipoles, was also validated using commercial full-wave software. The complex currents required for the shaping and obtained by the novel method were used to excite the full-wave models of the 49 element arrays. A very good agreement between the electric near-fields resulting from both the implemented algorithm and the full-wave calculations was obtained. In an important application scenario, it was shown that the method manages to target individual users who are distributed close to one another in the radiative near-field of the antenna array, as such ensuring the best quality of communication. The method has thus been validated via numerical and full-wave experiments, clearly demonstrating its applicability in (future) B5G and 6G real-time applications and communications scenarios.

Future plans include employing an accurate system impulse response to model applications defined in domains with specific shapes and symmetries. Moreover, an extension to 3D shaping is possible by making use of efficient optimization procedures owing to the algorithm’s impressive computation speed.

## Data Availability

The data presented in this study are available on request from the corresponding author.

## Appendix A. Green’s Function Multipole Expansion

The dyadic Green’s function G in (1) determines the electric field that an elementary point source, located at $r^{\prime }$, generates at the observation point r. It is defined as

(A1) $$G\left(r,r^{\prime }\right)=\left[I+\frac{\nabla \nabla }{k^{2}}\right]G\left(r,r^{\prime }\right),$$

in which I represents the unit dyadic, *k* is the wavenumber, and *G* is the scalar Green’s function, defined as

(A2) $$G\left(r,r^{\prime }\right)=-\frac{jk}{4\pi }h_{0}^{\left(2\right)}\left(k\left|r-r^{\prime }\right|\right),$$

where $h_{0}^{\left(2\right)}$ is the zeroth-order spherical Hankel function of the second kind ([38], p. 437). We now make use of

(A3) $$\nabla G\left(r,r^{\prime }\right)=-\nabla^{\prime }G\left(r,r^{\prime }\right),$$

where ∇ is the gradient performed with respect to r, while $\nabla^{\prime }$ represents the gradient performed with respect to $r^{\prime }$. By plugging (A3) in (A1) and by expanding (A2) according to the addition theorem for spherical Hankel functions ([43], Equation (2)), if r = *r* ≥ *R*, we obtain

(A4) $$G\left(r,r^{\prime }\right)=-jk\left[I+\frac{\nabla^{\prime }\nabla^{\prime }}{k^{2}}\right]\sum_{l,m}^{\infty }{\bar{J}}_{l}^{m}\left(kr^{\prime }\right)H_{l}^{m}\left(kr\right),$$

where the bar symbol indicates a complex conjugate. The multipole functions $J_{l}^{m}$ and $H_{l}^{m}$ are defined as

(A5) $$\begin{array}{cc} J_{l}^{m}\left(kr^{\prime }\right) & =j^{-l}j_{l}\left(kr^{\prime }\right)Y_{l}^{m}\left({\hat{r}}^{\prime }\right), \end{array}$$

(A6) $$\begin{array}{cc} H_{l}^{m}\left(kr\right) & =j^{-l}h_{l}^{\left(2\right)}\left(kr\right)Y_{l}^{m}\left(\hat{r}\right), \end{array}$$

where $j_{l}$ and $h_{l}^{\left(2\right)}$ are the $l^{\mathrm{th}}$ order spherical Bessel function of the first kind and spherical Hankel function of the second kind ([38], p. 437), respectively, and $Y_{l}^{m}$ denotes the scalar spherical harmonic [37]. By leveraging the plane wave expansion [49], we express the complex conjugate of (A5) as

(A7) $${\bar{J}}_{l}^{m}\left(kr^{\prime }\right)={\frac{1}{4\pi }}_{\Omega }e^{jk\cdot r^{\prime }}{\bar{Y}}_{l}^{m}\left(\hat{k}\right)d\hat{k},$$

which, plugged into (A4), yields the dyadic Green’s function’s multipole expansion (2) of Section 2, as

(A8) $$G\left(r,r^{\prime }\right)=-\frac{jk}{4\pi }\sum_{l,m}^{\infty }H_{l}^{m}\left(kr\right){∯}_{\Omega }\left[I-\hat{k}\hat{k}\right]e^{jk\cdot r^{\prime }}{\bar{Y}}_{l}^{m}\left(\hat{k}\right)d\hat{k}.$$
